# The relationship between the malignancy grade of lung adenocarcinoma with micropapillary pattern and the findings of positron emission tomography

**DOI:** 10.1186/1749-8090-10-S1-A152

**Published:** 2015-12-16

**Authors:** Norifumi Tsubokawa, Takahiro Mimae, Yasuhiro Tsutani, Takeshi Mimura, Yoshihiro Miyata, Morihito Okada

**Affiliations:** 1 Department of Respiratory Surgery, National Hospital Organization Kure Medical Centre and Chugoku Cancer Centre, Kure, Hiroshima, 737-002, Japan; 2 Department of Surgical Oncology, Hiroshima University, Hiroshima, 734-8551, Japan

## Background/Introduction

The survival rates are not always high after the complete resection even if early stage lung adenocarcinoma. Micropapillary pattern (MPP) was one of prognostic factors in such cancer.

## Aims/Objectives

This study aimed to investigate whether preoperative maximum standard uptake value (SUVmax) on positron emission tomography/computed tomography (PET/CT) could indicate early stage lung adenocarcinoma with MPP.

## Method

Total 347 consecutive patients with clinical stage IA lung adenocarcinoma that were treated by complete resection were retrospectively examined. We defined MPP- positive as accounting for 5 % or more of the entire tumor.

## Results

Forty eight (14%) and 299 (86%) patients were MPP-positive and negative, respectively. There were no significant differences between both groups in age (P = 0.369), gender (P = 0.059), or tumour size (P = 0.437). However, SUVmax on PET/CT were significantly higher in MPP-positive, than negative group (3.02 ± 2.34 vs. 2.19 ± 2.45, P = 0.029, respectively). In addition, lymphatic and vascular invasion as well as lymph node metastasis were more frequent in the MPP-positive, than negative group (P = 0.003, P = 0.029, and P = 0.002, respectively). Five-year recurrence free survival (RFS) rates were significantly lower in the MPP-positive, than negative group (69.7% vs. 89.3%, P < 0.001). Multivariate analysis for RFS showed that MPP, lymphatic and vascular invasion were independent poor prognostic factors (P = 0.048, P = 0.003, P = 0.002, respectively).

## Discussion/Conclusion

The presence (≤5%) of MPP in early stage lung adenocarcinoma should be considered a distinct subtype with a high risk of recurrence and a poor prognosis. In addition, preoperative PET/CT was useful for predicting whether tumours harboured MPP or not.

